# Mutation of *OsPIN1b* by CRISPR/Cas9 Reveals a Role for Auxin Transport in Modulating Rice Architecture and Root Gravitropism

**DOI:** 10.3390/ijms23168965

**Published:** 2022-08-11

**Authors:** Huihui Wang, Qiqi Ouyang, Chong Yang, Zhuoyan Zhang, Dianyun Hou, Hao Liu, Huawei Xu

**Affiliations:** College of Agriculture, Henan University of Science and Technology, Luoyang 471000, China

**Keywords:** *OsPIN1b*, pleiotropic phenotypes, polar auxin transport, gravitropism, rice (*Oryza sativa* L.)

## Abstract

The distribution and content of auxin within plant tissues affect a variety of important growth and developmental processes. Polar auxin transport (PAT), mainly mediated by auxin influx and efflux transporters, plays a vital role in determining auxin maxima and gradients in plants. The auxin efflux carrier PIN-FORMED (PIN) family is one of the major protein families involved in PAT. Rice (*Oryza sativa* L.) genome possesses 12 *OsPIN* genes. However, the detailed functions of *OsPIN* genes involved in regulating the rice architecture and gravity response are less well understood. In the present study, *OsPIN1b* was disrupted by CRISPR/Cas9 technology, and its roles in modulating rice architecture and root gravitropism were investigated. Tissue-specific analysis showed that *OsPIN1b* was mainly expressed in roots, stems and sheaths at the seedling stage, and the transcript abundance was progressively decreased during the seedling stages. Expression of *OsPIN1b* could be quickly and greatly induced by NAA, indicating that *OsPIN1b* played a vital role in PAT. IAA homeostasis was disturbed in *ospin1b* mutants, as evidenced by the changed sensitivity of shoot and root to NAA and NPA treatment, respectively. Mutation of *OsPIN1b* resulted in pleiotropic phenotypes, including decreased growth of shoots and primary roots, reduced adventitious root number in rice seedlings, as well as shorter and narrower leaves, increased leaf angle, more tiller number and decreased plant height and panicle length at the late developmental stage. Moreover, *ospin1b* mutants displayed a curly root phenotype cultured with tap water regardless of lighting conditions, while nutrient solution culture could partially rescue the curly root phenotype in light and almost completely abolish this phenotype in darkness, indicating the involvement of the integration of light and nutrient signals in root gravitropism regulation. Additionally, amyloplast sedimentation was impaired in the peripheral tiers of the *ospin1b* root cap columella cell, while it was not the main contributor to the abnormal root gravitropism. These data suggest that *OsPIN1b* not only plays a vital role in regulating rice architecture but also functions in regulating root gravitropism by the integration of light and nutrient signals.

## 1. Introduction

Auxin (mainly indole-3-acetic acid, IAA) is a universal phytohormone in plants, which requires polar transport and regulates various aspects of plant developmental and growth processes, such as embryogenesis, organogenesis, shoot elongation, root development, vascular tissue differentiation and tropisms [1,2]. Auxin is predominantly synthesized in the shoot apex and developing leaf primordia, and then transported to the targeted tissues by a polar auxin transport (PAT) system [3]. PAT is slower and regulated, occurring via carrier-mediated cell-to-cell directional transport [4]. PAT is established and maintained by the auxin transporters in plants: AUXIN/LIKE AUXIN (AUX/LAX) influx carriers, auxin efflux carriers of the PIN-FORMED (PIN) family, ATP-Binding Cassette subfamily B/P-glycoprotein (ABCB/PGP) subfamily of ABC transporters, and the newly described PIN-LIKES (PILS) proteins [2,5,6]. Directionality and rate of auxin movement are mainly controlled by asymmetric localization of the membrane-localized PIN proteins [7,8]. Rice (*Oryza sativa* L.), the most important staple food of over half of the world’s population [9,10,11], is considered one of the most suitable monocot models for plant molecular biology research because of the establishment of genome databases and mutant resources [12,13]. Extensive progress has been made in understanding the function of *AtPIN* genes in *Arabidopsis* [2,14]. However, the functions of *OsPIN* genes are still largely unknown. PIN1 is the first reported auxin efflux carrier that functions in shoot-basipetal auxin transport in *Arabidopsis,* and *pin1* mutants show a classical PIN-like inflorescence [15,16]. Thereafter, the *PIN* gene family has been extensively investigated in rice (*Oryza sativa* L.) [17,18,19], maize (*Zea mays* L.) [20,21,22], sorghum (*Sorghum bicolor*) [23], soybean (*Glycine max* L.) [24,25], cotton (*Gossypium hirsutum*) [26,27], potato (*Solanum tuberosum*) [28], Chinese cabbage (*Brassica rapa* L. ssp. pekinensis) [29], tomato (*Solanum lycopersicum*) [30], *Medicago truncatula* [31], *Populus* [32,33], switchgrass (*Panicum virgatum* L.) [34], olive (*Olea europaea*) [35], pear (*Pyrus pyrifolia*) [36] and coffee (*Coffea arabica*) [37].

There are 12 *PIN* genes in the rice genome: four *OsPIN1* (*OsPIN1a*-*OsPIN1d*), one *OsPIN2*, three *OsPIN5* (*OsPIN5a*-*OsPIN5c*), one *OsPIN8*, and three monocot-specific *PIN* genes, *OsPIN9*, *OsPIN10a*, and *OsPIN10b* [17,19]. To date, several *OsPIN* genes have been cloned and functionally characterized, such as the *OsPIN1* subfamily [3,7,38], *OsPIN2* [39,40,41,42,43,44,45], *OsPIN5b* [46] and *OsPIN10a* [47]. A previous study showed that rice *OsPIN1b* is involved in the regulation of adventitious root emergence and tiller number [3], while further investigation reported that *ospin1* single mutants had no dramatic phenotypes and only *pin1a pin1b* or *pin1c pin1d* double mutants showed obvious phenotypes, indicating the functional redundancy of *OsPIN1* genes in modulating rice architecture [7]. In addition, *OsPIN1b* is also involved in nitric oxide (NO) or strigolactones (SLs)-induced root elongation under low-nitrogen and -phosphate conditions [38]. *OsPIN2* is involved in root elongation growth, root gravitropic responses and lateral root formation patterns via regulating the basipetal auxin flow from the root tip towards the root-elongation zone [42,43,45]. OsPIN5b protein targets the endoplasmic reticulum and is involved in modulating tiller number, root system, panicle length and grain yield in rice [46]. *OsPIN9* is highly expressed in the vascular tissue of the root and stem base [19] and plays an important role in modulating adventitious root number and tiller number in rice [48]. *OsPIN10a*, also designated as *OsPIN3t*, is mainly expressed in vascular tissue and involved in the regulation of root growth and drought tolerance [47]. 

Although excellent progress has been made in understanding the underlying mechanisms of *OsPIN* genes involved in polar auxin transport in recent years, detailed information is still necessary to further dissect the role of *OsPIN* genes in regulating plant development and response to environmental cues. In this study, we reported the versatile role of *OsPIN1b* in regulating plant architecture and root gravitropism using *ospin1b* mutants created by CRISPR/Cas9 technology and further discussed the effects of different cultural conditions on *ospin1b* root gravitropism.

## 2. Results

### 2.1. Tissue-Specific Expression Pattern of OsPIN1b 

**Figure 1:**
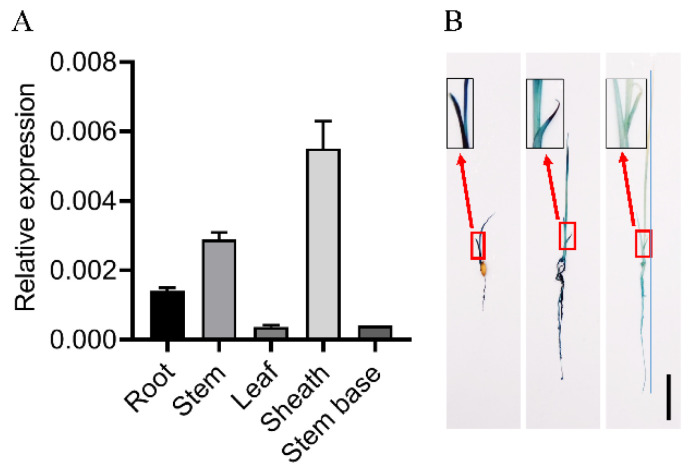
*OsPIN1b* expression in different tissues. (**A**) Tissue-specific expression pattern revealed by qRT–PCR. (**B**) GUS staining experiments in *OsPIN1b* promoter: *GUS* (*pOsPIN1b:GUS*) transgenic plants. Bar = 2 cm.

The tissue-specific expression pattern of *OsPIN1b* was investigated by qRT-PCR and β-glucuronidase (GUS) assay of transgenic plants expressing an *OsPIN1b* promoter:GUS (*pOsPIN1b:GUS*) fusion construct. qRT-PCR analysis showed that *OsPIN1b* mRNA was transcribed in all tissues tested in 14-day-old seedlings. High levels of *OsPIN1b* transcript were detected in the sheaths, and moderate levels were detected in the stems and roots, while relatively lower levels were found in the leaves and stem bases (Figure 1A). To confirm the tissue-specific expression of the *OsPIN1b* gene, the expression pattern of *OsPIN1b* was assayed by GUS staining. The results show that *OsPIN1b* is expressed to a relatively high level in early young seedlings and gradually decreases as the plants grow while still keeping a relatively high level in the roots (Figure 1B). 

### 2.2. Induction of OsPIN1b by Exogenous NAA and NPA

1-Naphthaleneacetic acid (NAA) can enter cells passively, and most of the identified *OsPIN* genes can respond to NAA treatment [7,46,47]. *N*-1-naphthylphthalamic acid (NPA) is one of the auxin transport inhibitors, which can be employed as a valuable tool to investigate auxin transporter-mediated developmental processes [49]. 

**Figure 2:**
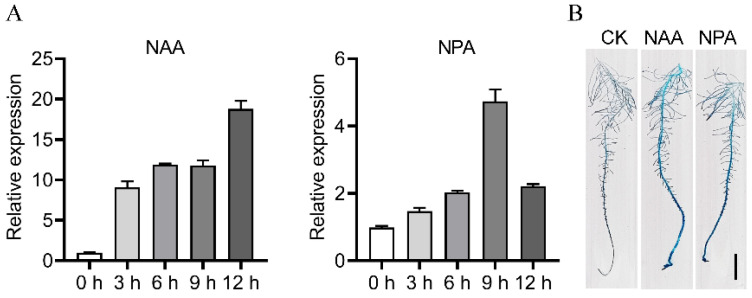
Expression analysis of *OsPIN1b* response to NAA and NPA treatment. (**A**) qRT-PCR analysis of *OsPIN1b* expression in rice seedling roots treated with NAA or NPA. (**B**) GUS staining experiment was performed after NAA or NPA treatment for 1 day. Bar = 0.5 cm.

To test the response of *OsPIN1b* to NAA or NPA treatment, we monitored the effects of NAA or NPA on the expression of *OsPIN1b* gene in rice roots by qRT-PCR. The 14-day-old seedlings were exposed to exogenous hormones of NAA at a concentration of 0.1 μM, or NPA at 0.5 μM. The results showed that *OsPIN1b* responded to exogenous NAA quickly and increased greatly after treatment for 3 h, the expression level was gradually increased, and the highest expression level was detected after treatment for 12 h (Figure 2A). For NPA treatment, with the treatment time increasing from 0 to 9 h, the transcript abundance increased gradually while decreasing after treatment for 12 h (Figure 2A).

To verify whether *OsPIN1b* is involved in polar auxin transport, 7-days seedlings of the *pOsPIN1b:GUS* transgenic plants were exposed to 0.1 µM NAA or 0.5 µM NPA, and GUS staining was performed after one-day treatment. Consistent with qRT-PCR results, GUS staining was darker after treatment with NAA or NPA, confirming the induction of *OsPIN1b* under NAA or NPA treatment (Figure 2B).

### 2.3. Generation and Identification of Rice ospin1b Mutants

**Figure 3:**
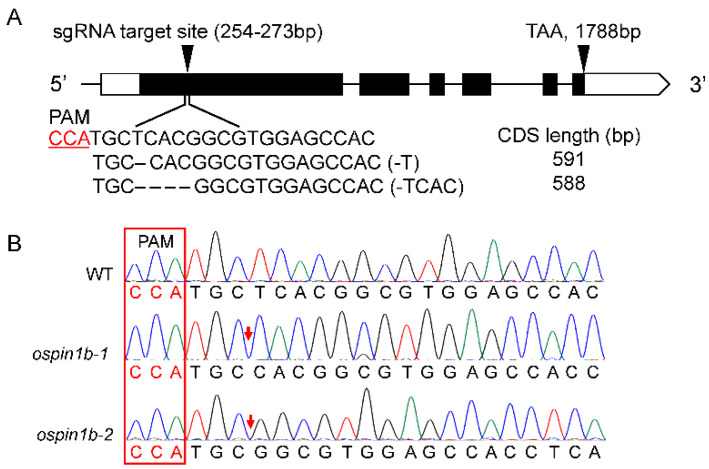
Schematic diagram of *OsPIN1b* gene mutation sites. (**A**) Gene structure of *OsPIN1b* and the sequence of the sgRNA target site. (**B**) A total of 1- and 4-bp nucleotides were deleted in the *ospin1b-1* and *ospin1b-2*, respectively. The red arrow shows the mutation site.

To further investigate the function of *OsPIN1b* in rice, two *ospin1b* mutants, designated as *ospin1b-1* and *ospin1b-2*, were generated using CRISPR/Cas9 technology. The *ospin1b-1* and *ospin1b-2* mutants contained a 1- and 4-bp deletion, respectively, in the first exon of *OsPIN1b* (Figure 3). The *OsPIN1b* mutation caused frame-shift mutation and premature termination of translation, and the mutation proteins contained 196 and 195 amino acids in *ospin1b-1* and *ospin1b-2* mutants, respectively. The function of PIN proteins is closely dependent on the number of transmembrane helices [50]. Bioinformatics analysis showed that only two transmembrane helices were present in the mutated OsPIN1b proteins, while the native OsPIN1b protein contains 10 transmembrane helices (Figure S1). These results indicate that *ospin1b* mutants lacked OsPIN1b. Off-target analysis confirmed that none of the off-target mutations was introduced into the *ospin1b* mutants (Figure S2). The progeny of these homozygous mutants was used for further study.

### 2.4. Auxin Homeostasis Is Disrupted in ospin1b Mutants

**Figure 4:**
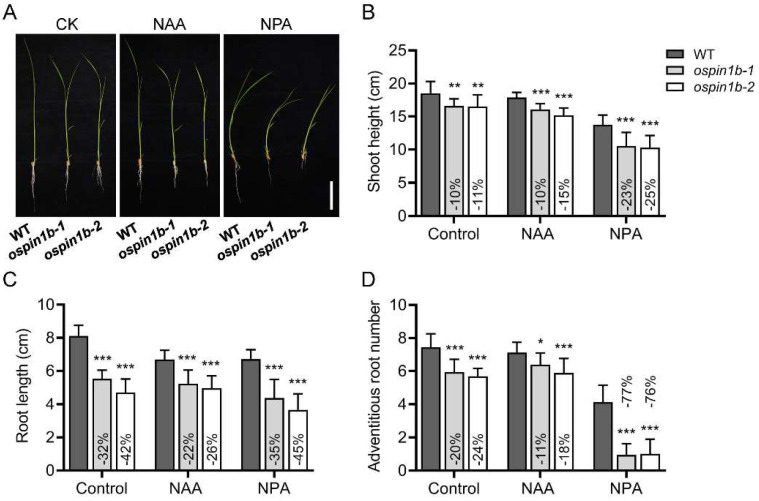
Response of *ospin1b* mutants to NAA or NPA treatment. (**A**) Phenotypes of wild-type (WT) and *ospin1b* mutants under normal, NAA or NPA treatments. Bar = 4 cm. (**B**) Statistical analysis of shoot height (**B**), root length (**C**) and adventitious root number (**C**) in WT and *ospin1b* mutants under normal, NAA or NPA treatments. Values are means ± standard deviation (**D**) (*n* = 24). Data were analyzed by ANOVA and Tukey’s tests. *: *p* < 0.05; **: *p* < 0.01; ***: *p* < 0.001.

Due to the fact that *OsPIN1b* can respond to NAA or NPA treatment (Figure 1), we further evaluated the response of *ospin1b* mutants to NAA or NPA treatment. Although the shoot height of *ospin1b* mutants under NAA treatment showed a similar decrease compared with that under normal conditions, NPA treatment greatly decreased the shoot height of *ospin1b* mutants (Figure 4A,B), indicating that *ospin1b* mutant shoots were more sensitive to NPA treatment. The root length of *ospin1b* mutants was reduced by 22–26% compared with wild-type (WT) plants under NAA treatment, while it decreased by 32–42% compared with WT plants under normal conditions, indicating that *ospin1b* roots were more tolerant to NAA treatment than WT roots (Figure 4C). Adventitious root development is tightly associated with auxin homeostasis [3,48]. Consistent with this view, adventitious root number was reduced by 20–24% compared to WT plants under normal conditions, while NPA treatment dramatically suppressed the emergence of adventitious roots, which was greatly decreased by 76–77%, in *ospin1b* mutants compared to that in WT plants (Figure 4D), indicating that *ospin1b* mutant roots were more sensitive to NPA treatment. Collectively, these data strongly illustrate that auxin homeostasis in *ospin1b* mutants is disturbed.

### 2.5. Mutation of OsPIN1b Results in Reduced Growth of Shoot and Primary Root, as well as Decreased Number of Adventitious Roots

**Figure 5:**
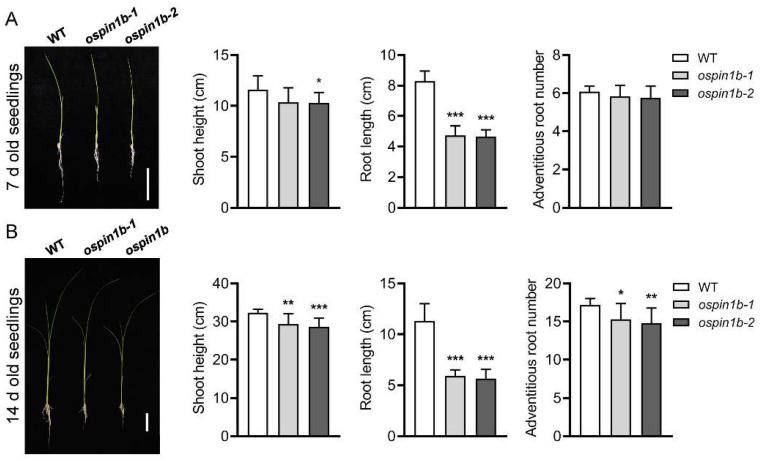
Phenotypes of wild-type (WT), *ospin1b-1* and *ospin1b-2* seedlings grown in the nutrient solution. (**A**) Phenotypes of 7-day-old seedlings. (**B**) Phenotypes of 14-day-old seedlings. Bar = 4 cm. Values are means ± standard deviation (SD) (*n* = 16). Data were analyzed by ANOVA and Tukey’s tests. *: *p* < 0.05; **: *p* < 0.01; ***: *p* < 0.001.

To understand the function of *OsPIN1b* at the seedling stage, the phenotypes of 7-day-old and 14-day-old seedlings were investigated. Compared with WT control, shorter primary roots of the *ospin1b* mutants were the most obvious phenotype. The primary root length was significantly decreased in comparison with the WT plants even after germination for only 7 days (Figure 5A). After germination for 14 days, the shoot height, root length and adventitious root number in *ospin1b* mutants were all significantly lower than that in WT plants (Figure 5B). These results show that the mutation of *OsPIN1b* significantly suppresses shoot and root growth and affects the development of adventitious roots.

### 2.6. Disruption of OsPIN1b Causes Shorter and Narrower Leaves and Larger Leaf Angle

**Figure 6:**
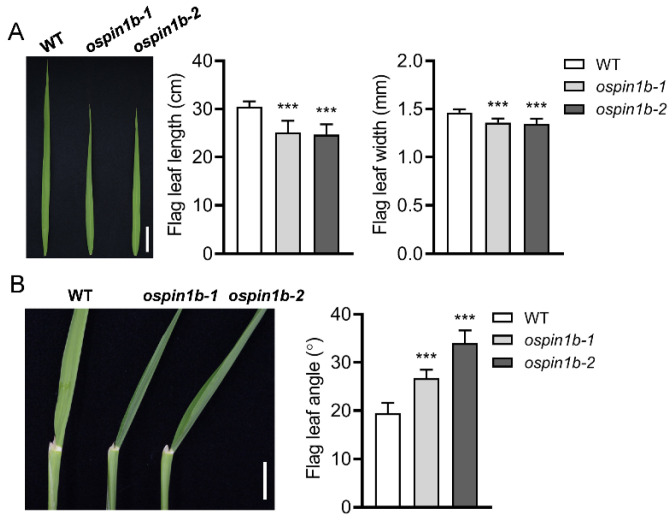
The phenotype of leaves in wild-type (WT) and *ospin1b* mutants at the heading stage. (**A**) Leaf length and width. Bar = 4 cm. (**B**) Leaf angle. Bar = 1 cm. Values are means ± standard deviation (SD) (*n* = 16). Data were analyzed by ANOVA and Tukey’s tests. ***: *p* < 0.001.

The 14-day-old seedlings were transferred into the soil and then cultured under normal conditions. Generally, *ospin1b* mutants grew more slowly than the WT plants, and the leaves of the *ospin1b* mutants were shorter and narrower than those of the WT plants. As shown in Figure 6A, the flag leaves of the *ospin1b* mutants were distinguished as shorter than that of the WT plants at the heading stage (90 days after germination), and the flag leaf length of the WT was about 30.5 cm on average, while it was about 25.0 cm in *ospin1b* mutants. The leaf width of *ospin1b* was significantly decreased in comparison with WT plants, which was 7–8% narrower than the WT plants (Figure 6A). Additionally, we also noticed that the flag leaf angle of *ospin1b* was larger than WT plants, which was significantly increased by 37.4% and 74.7% in *ospin1b-1* and *ospin1b-2* mutants, respectively, compared with WT plants (Figure 6B). These results suggest that *OsPIN1b* is involved in the regulation of leaf development.

### 2.7. Loss of function of OsPIN1b Leads to Decreased Plant Height, Increased Tiller Number, Reduced Panicle Length and Increased Grain Yield at the Mature Stage

**Figure 7:**
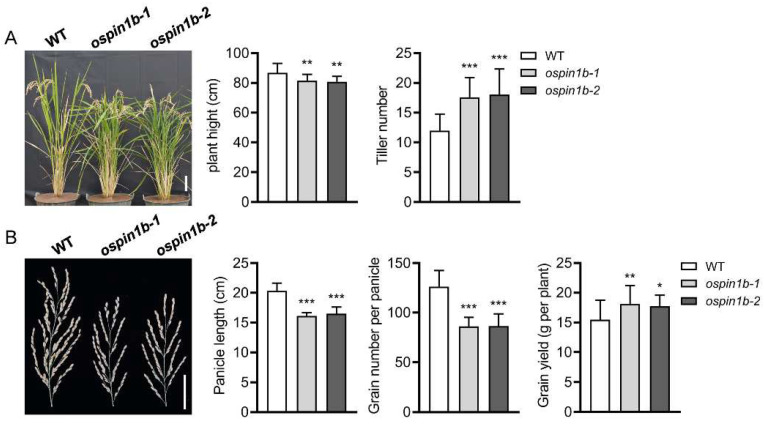
Phenotypes of wild-type (WT), *ospin1b-1* and *ospin1b-2* at the mature stage. (**A**) Plant height and tiller number. The plants were grown in the field and transferred to different plastic pots for photo shoots. Bar = 10 cm. (**B**) Panicle length, grain number per plant and grain yield per plant. Bar = 5 cm. Values are means ± standard deviation (SD) (*n* = 24). Data were analyzed by ANOVA and Tukey’s tests. *: *p* < 0.05; **: *p* < 0.01; ***: *p* < 0.001.

At the mature stage, the *ospin1b* mutants exhibited a clear phenotype, including decreased plant height, increased tiller number and shorter panicle compared with WT plants. The plant height of *ospin1b* mutants was significantly reduced by 6–7%, while the tiller number greatly increased by 47–51% compared with WT plants (Figure 7A). *ospin1b* mutants also had a decrease in the panicle length of about 20%, leading to a decrease in grain number per panicle relative to control plants of about 32% (Figure 7B). However, the grain yield per plant of *ospin1b* was significantly increased by 15–17% compared to wild-type plants, which was mainly attributed to the increased tiller number (Figure 7).

To verify which internode is attributed to the decreased plant height in *ospin1b* mutants, we further examined the lengths of the first to fifth internodes in WT and *ospin1b* mutants. The first internode length was significantly reduced in *ospin1b* mutants compared to WT plants, the second to fourth segments showed no significant difference from that of the WT plants, while the fifth internode length was significantly higher than that of the WT (Figure S3). These data suggest that the decreased plant height of *ospin1b* mutants is mainly caused by the decreased length of the first internode.

### 2.8. Mutation of OsPIN1b Alters Root Gravitropism

**Figure 8:**
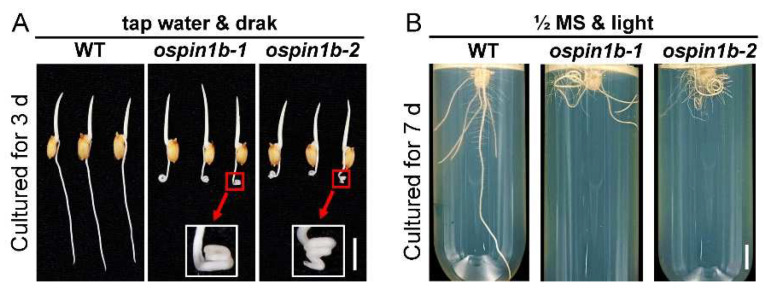
Mutation of *OsPIN1b* resulted in root agravitropism in *ospin1b* mutants. (**A**) Rice plants were cultured for 3 days with tap water in the dark. (**B**) Rice plants were cultured for 7 days with half-strength Murashige and Skoog (MS)-agar solid medium in light. Bar = 1 cm.

Root gravitropism is closely associated with auxin content and distribution within plant roots [51]. *OsPIN1b* is mainly expressed in root tips [19] and is probably involved in regulating root gravitropism. As expected, we noticed that *ospin1b* root tips exhibited a curly phenotype after germination for 3 days cultured in tap water and darkness (Figure 8A). We then further investigated the root phenotype cultured in half-strength Murashige and Skoog (MS)-agar solid medium in the light. As shown in Figure 8B, the roots of WT plants displayed a classical architecture with one primary root and several adventitious roots, while the roots of *ospin1b* lines lost the gravitropism and exhibited curly phenotype after being cultured for 7 days. These data strongly suggest that the mutation of *OsPIN1b* severely influences root gravitropism in rice.

### 2.9. The Root Agravitropism of ospin1b Mutants Is Influenced by Various Culture Conditions

**Figure 9:**
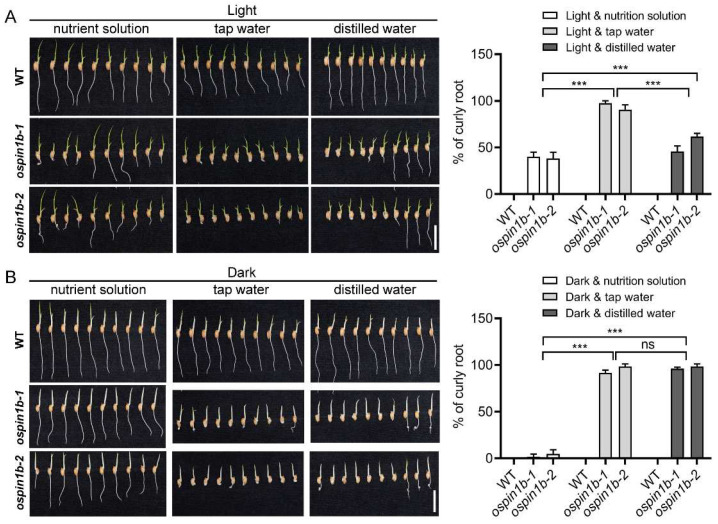
Root phenotype of wild-type (WT) and *ospin1b* mutants cultured under diverse conditions. (**A**) Rice plants were cultured with nutrient solution, tap water or distilled water in light. (**B**) Rice plants were cultured with nutrient solution, tap water or distilled water in the dark. Bar = 2 cm. Values are means ± standard deviation (SD) (*n* ≥ 36). Data were analyzed by ANOVA and Tukey’s tests. ***: *p* < 0.001.

Surprisingly, the curly root phenotype was not observed consistently. Further investigation showed that the curly root phenotype was tightly associated with culture conditions. *ospin1b* root showed a relatively higher curly root proportion when cultured with tap water or distilled water, especially when cultured with tap water, and almost all mutants displayed curly root phenotype, whereas a relatively lower curly root proportion was observed when cultured with the nutrient solution regardless of lighting conditions (Figure 9), indicating that culture conditions, especially the nutrient status, can indeed influence *ospin1b* root gravitropism. In addition to the nutrient conditions, lighting conditions also affected *ospin1b* root gravitropism. In light, about 40% *ospin1b* roots showed curly root phenotype cultured with nutrient solution (Figure 9A), while darkness almost completely abolished nutrient solution-grown *ospin1b* curly root phenotype (Figure 9B). In contrast, *ospin1b* mutant roots showed a relatively higher curly root proportion in the dark compared with that in light when both were cultured with distilled water (Figure 9A,B). These results demonstrate that light is also involved in regulating *ospin1b* root gravitropism.

### 2.10. Amyloplast Sedimentation Is Impaired in ospin1b Mutants, While It Is Not the Main Contributor to ospin1b Root Agravitropism

**Figure 10:**
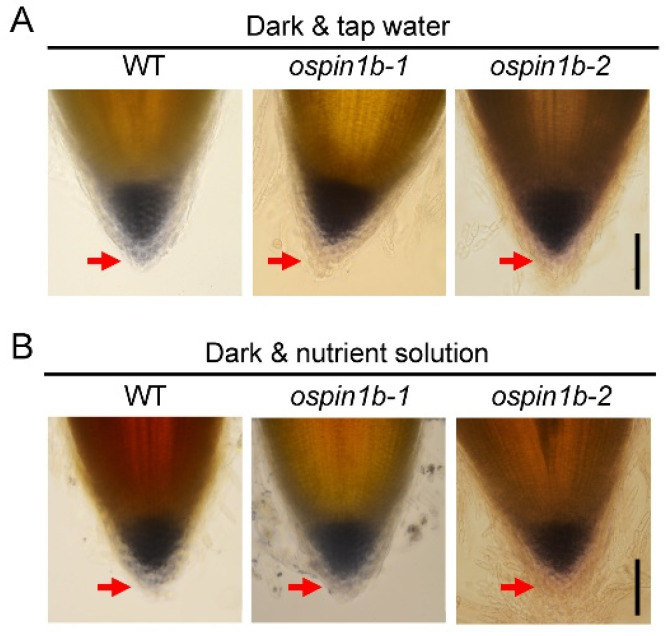
Amyloplast sedimentation analysis in wild-type (WT) and *ospin1b* root caps. (**A**) Rice plants were cultured with tap water in the dark. (**B**) Rice plants were cultured with a nutrient solution in the dark. The red arrow indicates the peripheral tiers of the root cap columella cell. Bar = 0.1 mm.

Evidence suggests that root gravitropism is tightly related to the starch content in root tips [52], and the sedimentation of starch-filled plastids (amyloplasts) in columella cells of the root cap is responsible for gravity sensing [53]. To determine whether *ospin1b* root agravitropism is associated with amyloplast sedimentation, we assayed the amyloplast sedimentation in tap water-grown rice root tips in darkness by light microscopy. Root caps of the WT and *ospin1b* plants were both stained intensely, and the staining was mainly restricted within columella cells. In contrast, the staining in *ospin1b* root caps was mainly detected in the inner tiers rather than the peripheral tiers of columella cell (Figure 10A), indicating that the amyloplast sedimentation is impaired in *ospin1b* root caps. 

For further assay as to whether the impaired amyloplast sedimentation in *ospin1b* root caps caused root agravitropism, we assayed the amyloplast sedimentation in *ospin1b* roots cultured with the nutrient solution in darkness, which showed a normal root phenotype. Staining results showed that there was no obvious difference in amyloplast sedimentation in nutrient solution-grown roots and tap water-grown roots (Figure 10B), indicating that the impaired amyloplast sedimentation is not the main contributor influencing *ospin1b* root gravitropism.

### 2.11. The Curly Root Phenotype in ospin1b Mutants Is Attributed to the Disturbance of Polar Auxin Transport and Intracellular Trafficking of OsPIN Proteins 

**Figure 11:**
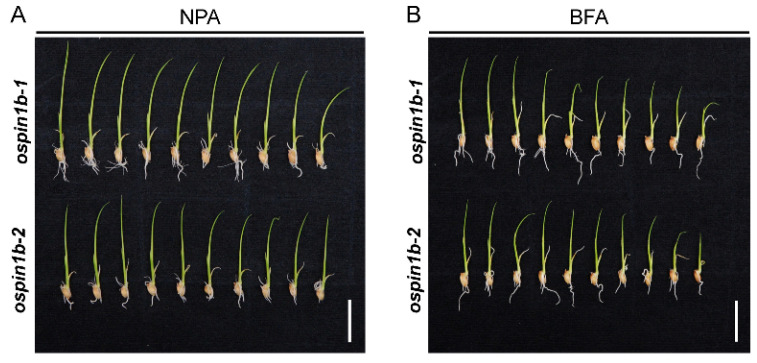
NPA and BFA treatment substantially rescued *ospin1b* curly root phenotype. (**A**) 0.01 μM NPA treatment. (**B**) 1 μM BFA treatment. Bar = 2 cm.

The asymmetric redistribution of auxin mediated by PIN carriers plays a vital role in modulating root development and gravitropism [53]. To dissect whether the *ospin1b* root agravitropism is caused by asymmetric redistribution of auxin mediated by PIN distribution and intracellular trafficking, we performed NPA and intracellular trafficking inhibitor fungal toxin brefeldin A (BFA) to treat *ospin1b* roots. NPA is an effective auxin transporter inhibitor [54], and BFA can specifically suppress vesicle trafficking [55]. Compared to the curly roots in tap water-grown *ospin1b* mutants, 0.01 μM NPA treatment partially rescued the curly root phenotype in *ospin1b* mutants (Figure 11A), while 1 μM BFA treatment almost completely abolished *ospin1b* curly root phenotype (Figure 11B). These results suggest that both the distribution and subcellular trafficking of OsPIN proteins are involved in regulating *ospin1b* root gravitropism. 

## 3. Discussion

Root development, which is closely associated with environmental parameters and phytohormones, is a fairly complex process. Although excellent progress has been made in root development for the model dicotyledon plant species *Arabidopsis thaliana*, the monocot model plant rice, characterized by a dense fibrous root system, still needs further dissection [56]. A variety of genes have been identified and functionally characterized in regulating rice root development [57], and almost all phytohormones are involved in modulating rice root development, among which auxin act as a central node to regulate root development communicated with other phytohormones [58]. In this present study, *ospin1b* mutants created by CRISPR/Cas9 technology were employed to systematically evaluate the phenotypic alteration at different development stages and environmental cues and nutrient status involved in regulating *ospin1b* root gravitropism were also dissected.

Auxin is necessary for almost all cellular processes, and an appropriate auxin level and distribution within plant tissues is associated with plant architecture determination [59,60]. *PIN* genes, which play a central role in auxin efflux, are closely related to auxin distribution, and disturbing the expression of *PIN* genes gives rise to abnormal architecture [61,62]. For example, mutation of *AtPIN1* causes the classical needle-like inflorescence in *Arabidopsis* [63], and disruption of *AtPIN4* affects root patterning [64]. 

The rice genome contains 12 *PIN* genes [19], and disturbing the expression of almost all *OsPIN* genes identified so far could impact rice architecture [3,7,39,46,47,48]. Other genes related to auxin synthesis, conjugation, storage, transport and catabolism also play a crucial role in regulating plant architecture [60,65,66,67,68,69]. One of the most important reasons is likely due to the disturbance of auxin homeostasis caused by the changed expression of these genes. A previous report has demonstrated that *OsPIN1b* is involved in auxin transport in rice [3]. Our results show that *OsPIN1b* is universally expressed in rice, implying that *OsPIN1b* might play a versatile role in rice development, especially in root development. We also noticed that *OsPIN1b* showed a strong expression in the coleoptile at the early seedling stage (Figure 1B), and further studies are needed to understand how *OsPIN1b* functions in the coleoptile at this developmental stage. Additionally, *OsPIN1b* can be quickly responsive to NAA and NPA treatment (Figure 2), indicating the involvement of *OsPIN1b* in auxin homeostasis. Further results showed that *ospin1b* mutant shoots and roots were both more sensitive to NPA treatment (Figure 4), indicating that the mutation of *OsPIN1b* disturbs the auxin homeostasis in rice plants. Consistently, *OsPIN1b* plays a critical role in orchestrating rice architecture, as evidenced by the pleiotropic phenotypes of *ospin1b* mutants at different development stages (Figure 5, Figure 6, Figure 7 and Figure 8). In addition to the shorter shoots and roots and reduced adventitious root number at the seedling stage (Figure 5), leave length and width, as well as leaf angle, were also changed in *ospin1b* mutants at the tilling stage (Figure 6). Mutation of *GmPIN1* genes also caused a change in leaf petiole angle in soybean (*Glycine max*) [70], indicating *PIN1* genes are likely to be involved in leaf angle regulation in plants. Consistent with the previous study [3], the tiller number of *ospin1b* mutants was also significantly increased compared with WT plants, and this change was concomitant with shorter panicles and, resultantly, decreased grain number per panicle at the mature stage, while the grain yield of *ospin1b* still increased significantly compared to WT plants (Figure 7), which was mainly attributed to the increased tiller number. Consistently, down-regulation of *TaPIN1s* also increased the tiller number and grain yield in wheat (*Triticum aestivum*) [71], suggesting that *PIN1* genes have the potential to regulate tiller number and grain yield in plants. Unexpectedly, we did not observe the larger tiller angle in *ospin1b* mutants, which was a clear phenotype in the RNAi plants [3]. It has been demonstrated that *OsLAZY1*, a gravitropism-related gene, regulates rice tiller angle by affecting the asymmetric redistribution of auxin [72,73], and overexpression of another auxin efflux carrier gene, *OsPIN2*, also increased rice tiller angle, which probably was mediated by suppressing the expression of *OsLAZY1* in the shoots [39], indicating tiller angle is closely associated with the expression of auxin transporters [74]. Collectively, the pleiotropic phenotypes of *ospin1b* mutants are reminiscent of the phenotype of OsIAAGLU-overexpressing transgenic rice, and these transgenic lines also showed decreased plant height, increased tiller number, larger leaf angle and root agravitropism [60]. 

*PIN* genes play a vital role in fine-tuning the content and distribution of auxin in various tissues [75,76], and auxin distribution is closely associated with root gravitropism [77]. Therefore, the proper polarization and expression levels of PIN proteins are necessary to maintain normal gravitropism in plants [77]. *ospin1b* mutants showed a curly root phenotype (Figure 8), indicating root gravitropism is impaired in *ospin1b* mutants. Similarly, disruption of the expression of *OsPIN2* also resulted in a curly root phenotype, and this phenotype could be almost abolished with NPA treatment, indicating that the curly root phenotype is closely related to polar auxin transport [42]. In line with this, we also noticed that NPA or BFA treatment could partially or almost completely abolish the curly root phenotype (Figure 11), emphasizing the involvement of polar auxin transport in modulating *ospin1b* root gravitropism. A recent report further confirmed that the wavy root phenotype, caused by the loss of function of *OsPIN2*, indeed resulted from the disrupted polar auxin transport in root tips, which then led to the alteration of auxin levels, auxin distribution and decreased amyloplast sedimentation in columella cells [45]. 

It was demonstrated that the first two tiers of root columella cells perceive the gravity stimulation [78] and transmit the signal to the zone of cell elongation, and the asymmetric accumulated auxin causes the gravitropic responses [79]. Conversely, the peripheral cap cells do not function in root gravity sensing [78]. In our observation, amyloplast sedimentation was decreased in peripheral tiers of *ospin1b* columella cells, whereas there was no obvious difference between nutrient solution-grown and tap water-grown *ospin1b* roots in the dark (Figure 10), indicating that the impaired amyloplast sedimentation in *ospin1b* root cap is not the main contributor to the curly root phenotype. Considering that *OsPIN1b* was highly expressed in root cap [19] and could be rapidly induced by NAA (Figure 2), it is reasonable to speculate that *OsPIN1b* is probably involved in the regulation of root gravity by transporting auxin, and this regulation might be disturbed in *ospin1b* root tips, which subsequently results in the agravitropism and curly root phenotype. 

It was reported that various culture conditions could influence *ospin2* root gravitropism [42]. Consistently, we also noticed that culture conditions can substantially influence *ospin1b* root gravitropism (Figure 9). The percentage of curly root in nutrient solution-grown *ospin1b* mutants under light conditions was significantly higher than that under dark conditions (Figure 9), indicating that lighting conditions are involved in regulating *ospin1b* root gravitropism. Given that auxin content in roots mainly mediated by polar auxin transport is closely associated with root gravitropism, we reasoned that light would improve the asymmetrical distribution of auxin in *ospin1b* roots, which ultimately increases the curly root proportion in nutrient solution-grown *ospin1b* mutants (Figure 9). In addition to light and dark, nutrient status can dramatically affect *ospin1b* root gravitropism. Almost all *ospin1b* showed a curly root phenotype cultured in tap water (Figure 9). By contrast, nutrient solution culture greatly rescued the curly root phenotype in *ospin1b* mutants, especially in darkness (Figure 9). Increasing evidence corroborated that nutrient status plays a dominant role in shaping root architecture, and auxin plays a central role in this process [80]. Different forms of nitrogen affect root growth and basipetal auxin transport, and this process is mainly mediated by different phosphorylation statuses and subcellular trafficking of PIN2 protein [81]. Moreover, the expression and subcellular trafficking of PIN2 are tightly associated with root gravitropism [53], and *OsPIN1b* also participates in regulating root elongation under low nitrogen and low phosphate conditions [38]. These results suggest that the expression, phosphorylation status, as well as subcellular trafficking of PIN carriers, which are closely associated with nutrient status, play a key role in regulating auxin transport and, correspondingly, root gravitropism. Collectively, root gravitropism is a much more complex biological process, which at least needs the cooperation of environmental cues and nutrient status to orchestrate different auxin carriers and, resultantly, the proper distribution of auxin in the root.

Taken together, OsPIN1b probably acts synergistically with OsPIN2 in regulating polar auxin transport and root gravitropism under different culture conditions in rice roots. Mutation of *OsPIN1b* impairs auxin homeostasis in rice roots and leads to the difference of lateral auxin gradient in root tips under diverse nutrient conditions and, resultantly, the differential curly root proportion. How the light and nutrient signals control root gravitropism is largely unknown, and we demonstrate that *ospin1b* mutants represent an excellent tool to study the action of light and nutrients as a signal molecule to regulate root gravitropism. Further functional studies are needed to determine the relationship between light, nutrients and root gravitropism.

## 4. Materials and Methods

### 4.1. Plant Materials and Growth Conditions

Rice *japonica* variety Nipponbare was used for the physiological experiments and rice transformation. For hydroponic experiments, the sterilized rice seeds were germinated in darkness for 3–4 days at 30 °C and then transferred to Kimura B complete nutrient solution [82] in plant growth chambers with 12-h-light (30 °C)/12-h-dark (25 °C) photoperiods, and the relative humidity was controlled at 60–70%. The initial pH of the solution was adjusted to 4.5–5.0, and the solution was replaced every 3 days. To evaluate the phenotypic traits at the seedling stage, rice plants were grown in plant growth chambers. To assay the agricultural traits at the mature stage, 14-day-old rice plants were transferred to soil and cultured under normal conditions. 

### 4.2. Vector Construction and Generation of the Transgenic Plants

The design of sgRNA targets, off-target analysis and mutation type identification were performed by the online toolkit CRISPR-GE (http://skl.scau.edu.cn/, accessed on 1 June 2017) [83]. A CRISPR/Cas9 system, CRISPR-RICE, which has been used successfully to create *ospin9* mutants, was employed to construct the *OsPIN1b*-editing recombinant vector [84]. For ligation of the synthesized DNA oligos to CRISPR-RICE vector, a TGTGT adapter and an AAAC adapter were added to the 5′ of the forward target sequence and reverse target sequence, respectively, and then a 4-nt overhang at the 5′ and 3′ ends of the oligo duplex was formed after annealing. CRISPR-RICE was digested with *Bsa* I firstly, and then the annealed oligos were inserted into CRISPR-RICE. The recombinant vector was designated as CRISPR-OsPIN1b.

The recombinant vector CRISPR-OsPIN1b was then transformed into *Agrobacterium tumefaciens* (EHA105) and employed for rice transformation according to the previous report [85]. 

### 4.3. Mutation Detection and Off-Target Analysis

The positive transgenic plants were firstly screened out by PCR using *HPT*-specific primers. Then a primer pair was designed to amplify genomic DNA fragments containing the sgRNA targeted sequence. The mutation types were then decoded by direct sequencing of PCR products [86]. Two homozygous *ospin1b* mutants, *ospin1b-1* and *ospin1b-2*, were identified and used for further research.

For off-target analysis, the potential off-target sites were first analyzed by CRISPR-GE, and the genotype of the targeted mutation regarding the top 3 potential off-target sites was further detected by sequencing the PCR amplicon directly [86]. 

### 4.4. GUS Staining

The *pOsPIN1b*:*GUS* transgenic rice was used for *OsPIN1b* tissue-specific analysis and NAA or NPA treatment experiments.

The histochemical detection of GUS activity was performed according to the previous report [87]. Briefly, transgenic plant samples were incubated with GUS reaction solution at 37 °C overnight. Green tissues were treated with ethanol before observation to remove the chlorophyll pigmentation. The stained tissues were photographed using a Nikon D7100 digital camera (Nikon Corporation, Tokyo, Japan).

### 4.5. Quantitative RT-PCR

qRT-PCR was performed according to our previous report [18]. In brief, total RNA was extracted from the samples of WT and *ospin1b* mutants using RNAiso Plus (Takara Bio Inc., Beijing, China). DNase I-digested RNAs were used for RT using HiScript III RT. SuperMix for qPCR (Nanjing Vazyme Biotech Company, Ltd., Nanjing, China). The gene-specific primers were designed using the online website INTEGRATED DNA TECHNOLOGIES (https://sg.idtdna.com, accessed on 1 January 2018). AceQ Universal SYBR qPCR Master Mix (Nanjing Vazyme Biotech Company, Ltd., Nanjing, China) and Lightcycle^®^ 96 system were employed for qRT-PCR. Three biological replicates and three technical repetitions were performed to assay the gene expression, and *OsACTIN1* gene (Os03g0718100) was used as an internal control. All primers used in this study are given in Table S1.

### 4.6. Exogenous NAA and NPA Treatment

For analysis of the response of *OsPIN1b* to NAA and NPA treatment, 14-day-old seedlings were transferred into Kimura B complete nutrient solution containing 0.1 μM NAA or 0.5 μM NPA for 0, 3, 6, 9 and 12 h. Roots were collected and used for qRT-PCR analysis.

For NAA and NPA sensitivity experiments, germinated rice seeds of WT and *ospin1b* mutants were transferred to Kimura B complete nutrient solution containing 0.01 μM NAA or 0.25 μM NPA and grown for 7 days, and then the shoot height, root length and adventitious root number were assessed. WT and *ospin1b* mutants cultured under normal conditions were employed as a control in this study.

### 4.7. Amyloplast Sedimentation Experiment

WT and *ospin1b* seeds were germinated for 2–3 days in tap water and darkness, and then the root tips were sampled for amyloplast sedimentation observation. To observe the amyloplasts in the columella cells of the root cap, roots were soaked and stained with I_2_-KI solution (0.33% (*w*/*v*) I_2_ and 0.67% (*w*/*v*) KI) for 5 min. Then the root tips were rinsed in chloral hydrate (40 g chloral hydrate was dissolved in 10 mL glycerine and 20 mL ddH_2_O) for 2–6 min. The root tips were observed under the microscope.

### 4.8. Phenotypic Analysis and Root Gravitropism Evaluation

Plant height, leaf length and width, and root length were measured using a ruler. Leaf angles between the sheath and flag leaf after germination for 90 days were photographed and measured with ImageJ.

For root gravitropism analysis, WT and *ospin1b* mutants were cultured in Kimura B complete nutrient solution, tap water or distilled water for 2–3 days in light or darkness. For curly root phenotype observation, the seeds of WT and *ospin1b* mutants were sowed on the surface of half-strength Murashige and Skoog (MS)-agar solid medium for 7 days in the light.

BFA is a valuable tool for deciphering the mechanisms of the polarization of PIN [88]. BFA treatment was performed to study the effect of intracellular trafficking on *ospin1b* root gravitropism. Germinated seeds were transferred into tap water containing 1 μM BFA and cultured for 4 days, and then the percentage of the curly root was assayed.

To evaluate the effect of polar auxin transport on *ospin1b* root gravitropism, germinated seeds were transferred into tap water containing 0.01 μM NPA and cultured for 5 days, and then the curly root proportion was analyzed. 

### 4.9. Data Analysis

All physiological experiments were repeated at least three times with consistent results. Experimental data were statistically analyzed by one-way analysis of variance (ANOVA) method in GraphPad PRISM (8.0.2) at the significance levels of *p* < 0.05 (*), *p* < 0.01 (**) and *p* < 0.001 (***), and all data are displayed as means ± SD.

## 5. Conclusions

Mutation of *OsPIN1b* causes pleiotropic phenotypes at different developmental stages, and *ospin1b* root gravitropism is cooperatively regulated by the involvement of lighting conditions and nutrient status.

## Data Availability

Not applicable.

## Supplementary Materials

The following supporting information can be downloaded at: https://www.mdpi.com/article/10.3390/ijms23168965/s1.
