# Supplementary material for: Mutation of OsPIN1b by CRISPR/Cas9 Reveals a Role for Auxin Transport in Modulating Rice Architecture and Root Gravitropism
Source: Int J Mol Sci. 2022 Aug 11;23(16):8965. doi: 10.3390/ijms23168965 (PMC9409181; doi:10.3390/ijms23168965)
Supplement: Supplementary file 1 [file ijms-23-08965-s001.zip › ijms-1844028-Supplementary.pdf]

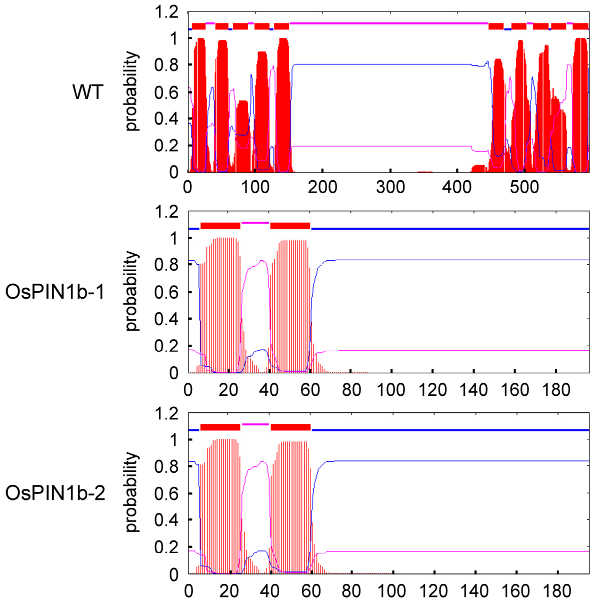

Figure S1. Transmembrane helices analysis of OsPIN1b proteins in wild-type (WT) and *ospin1b* mutants. The transmembrane helice analysis was performed by TMHMM-2.0: https://services.healthtech.dtu.dk/service.php?TMHMM-2.0, and the red peaks indicate the predicted transmembrane helices of proteins.

A

Chrom	Position	Sequence	Off-score	Gene	Region
chr02	31161577	GTGGCTCCACGCCGTGAGCA TGG	1	OS02G0743400	CDS
chr01	7625250	TTGGCAACATGCCGTGTGCA TGG	0.082		intergenic
chr02	29438985	GTGCCTCCGCGCCCTGACCA CGG	0.072	OS02G0710700	CDS
chr04	34335020	GTGGCTACATGCAGAGAGGA TGG	0.02	OS04G0672700	CDS
chr02	4946862	CGCGCTCCACGTCGTGAGCA TCG	0.013	OS02G0189300	CDS
chr12	17981895	GTAGCTCCACACCCAGAGCA TGA	0.013	OS12G0485600	intron
chr02	32463546	GTGGCTGCACGCCGTGAGCA CGC	0.01	OS02G0769900	CDS
chr06	13539068	GTGGCTGCACGTCCTGTTCA TGG	0.01		intergenic
chr07	23797357	GTGGCTCGACGCCGTGGCCA CCG	0.006	OS07G0585700	CDS
chr07	4445194	GTTGCTCCAAGCAGTGAGCA AGC	0.004		intergenic
chr09	2502497	GTGCCTCCCCGCCGAGCGCA TCG	0.003	OS09G0132900	CDS
chr04	31782018	GTGCTTCCACGCCGTGCGCT TGC	0.001		intergenic

B

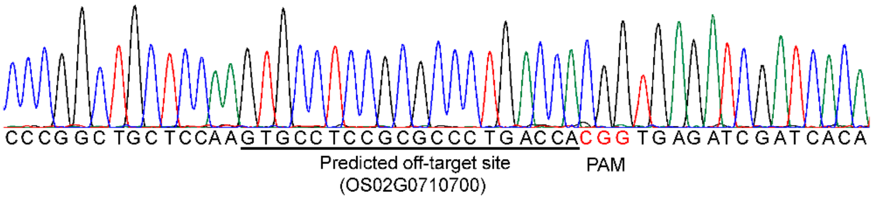

Figure S2. Off-target analysis of *ospin1b* mutants. (A) The potential off-target sites predicted by CRISPR-GE. (B) The sequencing chromatogram of the predicted potential off-target site.

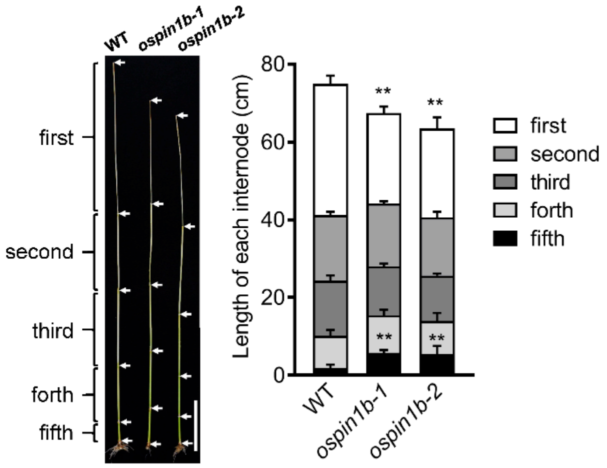

Figure S3. Internode length of wild-type (WT) and *ospin1b* mutants at the mature stage. Bar = 10 cm. Values are means ± standard deviation (SD) (n = 16). Data were analyzed by ANOVA and Tukey's tests. **: $p < 0.01$.

Table S1. Primers used in this study.

**Primers for vector construction**	Primer sequences (5'-3')
OsPIN1b-CRISPR-F	TGTGTGTGGCTCCACGCCGTGAGCA
OsPIN1b-CRISPR-R	AAACTGCTCACGGCGTGGAGCCAC
**Primers for screening of the transgenic plants**	
HPT-F	CTGAACTCACCGCGACGTCTGTC
HPF-R	TAGCGCGTCTGCTGCTCCATACA
**Primers for mutant identification**	
PIN1b-Assay-F	GTGAAATAGTGCCACCGAGTGAGCGC
PIN1b-Assay-R	CCATGAGGCTGCCGGAGAACTCC
**Primers for off-target analysis**	
700-OT-F	TTCTGCTACTGCGGGGTGAGG
700-OT-R	TAGGGGAATGGAGGCATGTTC
**Primers for qRT-PCR**	
OsPIN1b-qF	GAATCGTGCCCTTTGTGTTTG
OsPIN1b-qR	TGTAGTAGACGAGGGTGATAGG
OsACTIN1-qF	CTTCATAGGAATGGAAGCTGCG
OsACTIN1-qR	CACCTTGATCTTCATGCTGCTA
